# Coronary Artery Bypass Grafting Using the No-Touch Great Saphenous Vein Graft Harvesting Technique: A Retrospective Study

**DOI:** 10.7759/cureus.50777

**Published:** 2023-12-19

**Authors:** Tomohiro Nakajima, Tsuyoshi Shibata, Shuhei Miura, Kei Mukawa, Takakimi Mizuno, Keitaro Nakanishi, Ayaka Arihara, Junji Nakazawa, Yutaka Iba, Nobuyoshi Kawaharada

**Affiliations:** 1 Cardiovascular Surgery, Sapporo Medical University, Sapporo, JPN

**Keywords:** surgical site infection (ssi), patent, great saphenous vein, internal thoracic artery harvesting site, cabg surgery

## Abstract

Background

We focused on coronary artery bypass grafting using the great saphenous vein and compared the no-touch great saphenous vein and conventional great saphenous vein.

Methods

Coronary artery bypass grafting using the great saphenous vein was performed at our hospital over a 15-year period from 2007/04 to 2022/08. The primary endpoint was the patency of the great saphenous vein at discharge, and secondary endpoints were delayed healing of the great saphenous vein harvest wound, delayed healing of the mid-thoracic wound, and factors related to coronary artery bypass surgery.

Results

There were 183 patients who underwent coronary artery bypass surgery using the great saphenous vein during the study period. There were 131 male patients (72%) and 52 female patients (28%) with a mean age of 69 years (38-94 years). The method of harvesting the great saphenous vein was a no-touch great saphenous vein graft (NT-SVG) in 29 cases (16%) and conventional SVG in 154 cases (84%). Patients were divided into two groups: the NT-SVG group and the standard-collection saphenous vein graft (SVG) group. We compared graft patency at discharge, healing failure of the lower leg wound, healing failure of the mid-thoracic wound, and flow by transit-time flow measurement (TTFM).

Conclusion

There were no significant differences in perioperative outcomes between the NT-SVG and conventional SVG groups in this study.

## Introduction

Venous grafts are receiving renewed attention as the second graft of choice after the internal thoracic arteries (ITAs) for coronary artery bypass grafting (CABG) [1, 2]. Research has shown that the no-touch method, in which the venous graft is harvested with pedicles, improves the patency rate [3]. In this study, we compared the mid-term results of a no-touch great saphenous vein graft (NT-SVG) harvesting method with the conventional saphenous vein graft (SVG) harvesting method [4] for CABG in our department. This article was previously presented as a meeting abstract at the 2023 International Society for Minimally Invasive Cardiothoracic Surgery Annual Scientific Meeting on 31 May, 2023.

## Materials and methods

Study population

CABG using the great saphenous vein (GSV) was performed at Sapporo Medical University hospital over a 15-year period from April 2007 to August 2022.

Analyzed factors and primary endpoint

The primary endpoint was the patency of the GSV at discharge. The secondary endpoints were delayed healing of the GSV harvest wound, delayed healing of the mid-thoracic wound, and factors related to CABG.

Strategy of CABG

Complete revascularization is the basic procedure in CABG. The bilateral ITAs are used, and the remaining anastomosis is performed using the GSV, gastric aorta, and radial artery. The bilateral ITAs are used even in patients with diabetes mellitus.

Definitions of surgical site infection

Superficial and deep incisional surgical site infection (SSI) were evaluated in this study. Superficial incisional SSI is localized to the skin area where the surgical incision was made. Deep incisional SSI extends beyond the skin and affects deeper tissues, including muscles and the surrounding tissues beneath the incision area.

Statistical methods

Continuous variables are reported as mean ± standard deviation. Categorical variables are presented as raw numbers (percentage) and were compared using the χ2 test and Fisher’s exact test. All calculations were performed using JMP version 17 (SAS Institute, Inc., Cary, NC, USA).

## Results

Patient characteristics

In total, 183 patients were enrolled. Their mean age was 69.2 ± 9.4 years, and 131 (72%) were male. Their average body surface area was 1.65 m^2^. Prevalent comorbidities included hypertension in 80 (43%) patients, hyperlipidemia in 58 (31%), diabetes treated with oral antidiabetic therapy in 44 (24%), and diabetes treated with insulin in 32 (17%). Smoking was reported in 118 (64%) patients, and the preoperative logistic EuroSCORE was 7.2. These results are shown in Table 1.

**Table 1:** Demographics and comorbidities Categorical data are presented as number (%) and continuous data as mean ± standard deviation

Variables	n=183
Age, years	69.2 ± 9.4
Male	131 (72)
Body surface area, kg/m^2^	1.65 ± 0.20
Hypertension	80 (43)
Dyslipidemia	58 (31)
Diabetes mellitus(oral antidiabetic drugs)	44 (24)
Diabetes mellitus (insulin)	32 (17)
Smoking	118 (64)
Logistic EuroSCORE	7.2 (0.8-68.3)

Operative characteristics

The mean number of peripheral anastomoses was 3.0 ± 1.4. The bilateral ITAs were used as grafts in 76 (42%) patients, the left ITA in 133 (73%), the right ITA in 79 (43%), the gastroepiploic artery (GEA) in 14 (8%), and the GSV in all 183 (100%). The NT-SVG harvesting technique was used in 29 (16%) patients, and the conventional SVG harvesting technique was used in 154 (84%). Off-pump CABG was performed in 85 (44%) patients, on-pump beating CABG in 12 (7%), and on-pump arrest CABG in 86 (47%). Elective surgery was performed in 152 (83%) patients, and emergency surgery was performed in 31 (17%). In terms of surgical techniques, isolated CABG was performed in 98 (54%) patients, CABG combined with valve surgery in 63 (34%), CABG combined with aortic surgery in 14 (8%), and CABG combined with aortic dissection in 8 (4%). Unplanned CABG procedures occurred in 8 (4%) patients. The average surgical duration was 482 minutes. These results are presented in Table 2.

**Table 2:** Procedure Characteristics Categorical data are presented as number (%) NT-SVG - no-touch saphenous vein graft, SVG - saphenous vein graft, CABG - coronary artery bypass graft, ITA - internal thoracic artery, LITA - left internal thoracic artery, RITA - right internal thoracic artery, GEA - gastroepiploic artery

Variables		Overall (N = 183)
No. of distal anastomoses per patient		3.0± 1.4
Use of graft for CABG		
Bilateral ITA		76 (42)
LITA		133 (73)
RITA		79 (43)
GEA		14 (8)
Saphenous vein		183 (100)
NT-SVG		29 (16)
Conventional SVG		154 (84)
Off-pump CABG		85 (46)
On-pump beating CABG		12 (7)
On-pump arrest CABG		86 (47)
Urgency		
Elective		152 (83)
Emergent		31 (17)
Procedure		
Isolated CABG		98 (54)
CABG+valve surgery		63 (34)
CABG+Aorta		14 (8)
Aortic dissection+CABG		8 (4)
Unplanned CABG		8 (4)
Operation time of open repair (min)		482 ± 137

Postoperative findings

The postoperative results are shown in Table 3, which stratifies the analysis into two groups: the NT-SVG and conventional SVG harvesting techniques. There was no significant difference in the postoperative patency rate of the GSV graft between the two groups (p=0.64). Transit-time flow measurement also showed no significant difference between the NT-SVG group (27 mL/min) and the conventional SVG group (38 mL/min) (p=0.31). The incidence rates of superficial SSI (p=0.29) and deep SSI (p=0.63) at the GSV harvesting site showed no significant differences between the two groups. Similarly, there were no significant differences in the occurrence of superficial SSI (p=0.20) and mediastinitis (p=0.71) at the midline chest incision site.

**Table 3:** Comparison of NT-SVG with conventional SVG NT-SVG - no-touch saphenous vein graft, SVG - saphenous vein graft, SSI - surgical site infection, TTFM - transit time flow meter

Variable	NT-SVG (n=29)	Conventional SVG (n=154)	p-value
Graft patency at discharge	27 (93%)	143 (93%)	0.64
TTFM flow (ml/min)	27	38	0.31
Superficial SSI at lower extreme	4 (14%)	12 (8%)	0.29
Deep SSI at lower extreme	1 (3%)	7 (5%)	0.63
Superficial SSI at thoracic lesion	0	9 (6%)	0.20
Mediastinitis	0	2 (1%)	0.71

## Discussion

In CABG, the long-term patency of the ITA has been extensively reported as favorable [5]. Moreover, no significant differences in long-term patency have been reported between the left and right ITA [6]. When multiple anastomoses are required in CABG, surgeons consider graft options other than the ITA, such as the GSV, radial artery, or gastroepiploic artery [7]. However, the long-term patency of these grafts is inferior to that of the ITA [8]. Therefore, various strategies have been employed to enhance the long-term patency of these vessels, with one notable method being the preservation of adipose tissue during the harvesting of the GSV.

Avoiding contact with the GSV and not pre-expanding the vein before anastomosis was historically believed to contribute to its long-term patency. In recent years, studies have suggested that the adipose tissue surrounding the GSV contains mediators that inhibit vascular degeneration, potentially contributing to improved long-term patency [9]. However, in lower limb GSV harvesting, where the vein runs slightly dorsal to the tibia and has relatively little subcutaneous fat, concerns about wound healing impairment arise. This is particularly true with the NT-GSV harvesting technique, which includes concurrent fat tissue extraction.

In the present study, we retrospectively examined whether there were differences in the patency rates, intraoperative flow, and wound healing delays between the NT-SVG and conventional SVG harvesting techniques, and no statistically significant differences were found. The variability in the amount of fat attached during NT-SVG harvesting, depending on the individual harvester, has led to an absence of strict definitions for this technique. Further research focusing on the amount of fat attached to the GSV and its relationship to long-term graft patency is anticipated.

## Conclusions

We investigated the surgical outcomes of CABG using the GSV in 183 patients at our institution. The patients were divided into two groups based on the graft harvesting technique used: the NT-SVG harvesting group and the conventional SVG harvesting group. We found no significant differences in early postoperative patency rates or flow between the two groups. There were also no significant differences in the occurrence of superficial SSI or mediastinitis associated with the midline chest incision between the two groups. Because of the adherence of adipose tissue during harvesting, we anticipated delayed wound healing in the lower extremity in the NT-SVG group. However, there was no significant difference in lower extremity wound healing. Therefore, we conclude that if the NT-SVG harvesting technique demonstrated an equal long-term prognosis as a conventional saphenous vein graft, it can be chosen without concerns regarding wound healing complications.
